# Patient Experience and Satisfaction in Online Reviews of Obstetric Care: Observational Study

**DOI:** 10.2196/28379

**Published:** 2022-03-31

**Authors:** Emily K Seltzer, Sharath Chandra Guntuku, Amy L Lanza, Christopher Tufts, Sindhu K Srinivas, Elissa V Klinger, David A Asch, Nick Fausti, Lyle H Ungar, Raina M Merchant

**Affiliations:** 1 Penn Medicine Center for Digital Health University of Pennsylvania Philadelphia, PA United States; 2 Penn Medicine Center for Health Care Innovation University of Pennsylvania Philadelphia, PA United States; 3 Department of Computer and Information Science University of Pennsylvania Philadelphia, PA United States; 4 Department of Obstetrics and Gynecology University of Pennsylvania Philadelphia, PA United States; 5 Center for Health Equity Research and Promotion Corporal Michael J. Crescenz VA Medical Center Philadelphia, PA United States

**Keywords:** patient satisfaction, Yelp, online reviews, labor and delivery, ob-gyn, quality improvement, machine learning, labor, delivery, natural language processing, maternal health, ML, patients, obstetrics

## Abstract

**Background:**

The quality of care in labor and delivery is traditionally measured through the Hospital Consumer Assessment of Healthcare Providers and Systems but less is known about the experiences of care reported by patients and caregivers on online sites that are more easily accessed by the public.

**Objective:**

The aim of this study was to generate insight into the labor and delivery experience using hospital reviews on Yelp.

**Methods:**

We identified all Yelp reviews of US hospitals posted online from May 2005 to March 2017. We used a machine learning tool, latent Dirichlet allocation, to identify 100 topics or themes within these reviews and used Pearson *r* to identify statistically significant correlations between topics and high (5-star) and low (1-star) ratings.

**Results:**

A total of 1569 hospitals listed in the American Hospital Association directory had at least one Yelp posting, contributing a total of 41,095 Yelp reviews. Among those hospitals, 919 (59%) had at least one Yelp rating for labor and delivery services (median of 9 reviews), contributing a total of 6523 labor and delivery reviews. Reviews concentrated among 5-star (n=2643, 41%) and 1-star reviews (n=1934, 30%). Themes strongly associated with favorable ratings included the following: top-notch care (*r*=0.45, *P*<.001), describing staff as comforting (*r*=0.52, *P*<.001), the delivery experience (*r*=0.46, *P*<.001), modern and clean facilities (*r*=0.44, *P*<.001), and hospital food (*r*=0.38, *P*<.001). Themes strongly correlated with 1-star labor and delivery reviews included complaints to management (*r*=0.30, *P*<.001), a lack of agency among patients (*r*=0.47, *P*<.001), and issues with discharging from the hospital (*r*=0.32, *P*<.001).

**Conclusions:**

Online review content about labor and delivery can provide meaningful information about patient satisfaction and experiences. Narratives from these reviews that are not otherwise captured in traditional surveys can direct efforts to improve the experience of obstetrical care.

## Introduction

Many hospitals in the United States use the Hospital Consumer Assessment of Healthcare Providers and Systems (HCAHPS) and Press Ganey surveys to evaluate patient experiences [1]. Survey results are standardized and publicly reported to facilitate comparisons of patient experience. However, they are costly and often have low response rates [2,3]. Prespecified domains may miss the concerns of many patients, and aggregated public reporting can obscure differences across specialties [4,5].

Yelp is a website where users share information about their experiences at local businesses by giving a star rating from 1-5 and leaving a narrative review. Yelp is the most used free website in the United States for hospital ratings [6]. In one study, 65% of gynecologists reported being likely to use online ratings to improve patient care, more so than physicians from other specialties [7]. Prior work demonstrates that reviews from online rating services like Yelp are correlated with traditional methods for understanding the patient experience, and the platform’s unstructured design provides information not captured in conventional patient experience surveys [2,8-12]. The scale and utilization of these platforms is significant and may provide a nuanced way to better listen to patients [13]. In this study, we aim to evaluate how the content of labor and delivery Yelp reviews relates to star rating to provide insight into the labor and delivery experience in the United States.

## Methods

### Obtaining Hospital Reviews

We identified hospitals in the United States that have Yelp reviews using the Yelp Search application programming interface. We included only hospitals listed in the American Hospital Association directory with at least one review. Hospital reviews were then searched for keywords specific to labor and delivery, identified by referencing the Unified Medical Language System database and gathering input from an obstetrician (SKS). The search terms included variations of the same word—for example, “deliver” and “delivery” were both used but counted as one search term. Reviews containing at least one of the specified keywords were characterized as “labor and delivery reviews” and all others as “non–labor and delivery” (Table 1). We used only reviews that received a 5-star or 1-star review for the final analyses, considering the bimodal distribution (Table 2).

**Table 1:** Characteristics of hospitals with labor and delivery Yelp reviews.

Characteristic			All hospitals on Yelp (N=1569)				All labor and delivery hospitals on Yelp (N=919)		
			Hospitals, n (%)		Average Yelp rating (on a scale from 1-5)		Hospitals, n (%)		Average Yelp rating (on a scale from 1-5)
**Bed size**									
	0-49	223 (14)		3.23		46 (5)		3.44	
	50-199	577 (37)		2.86		329 (36)		3.31	
	200-399	487 (31)		2.84		334 (36)		3.24	
	≥400	275 (18)		2.88		210 (23)		3.33	
**Region**									
	Northeast	305 (20)		2.84		191 (21)		3.16	
	South	617 (40)		2.90		330 (36)		3.23	
	Midwest	184 (12)		3.12		71 (8)		3.45	
	West	456 (29)		2.88		327 (36)		3.35	
**Teaching hospital**									
	Yes	192 (12)		2.92		157 (17)		3.27	
	No	1370 (88)		2.85		762 (83)		3.29	

**Table 2:** Distribution of ratings of hospital reviews describing labor and delivery versus all hospital reviews.

Rating (stars)	Labor and delivery Yelp reviews (N=6523), n (%)	Hospital Yelp reviews (N=41,095), n (%)
1	1957 (30)	16,849 (41)
2	522 (8)	3288 (8)
3	457 (7)	2466 (6)
4	913 (14)	5342 (13)
5	2674 (41)	13,150 (32)

### Deriving Language Features

After removing stop words, common words of low information content (eg, “the,” “as,” “a”), we used the MALLET implementation [14] of the machine learning program latent Dirichlet allocation (LDA) to generate 100 topics based on prior work [13]. This machine learning technique automates the identification of co-occurring words whose combination suggests themes or topics [15]. For example, the frequent co-occurrence of “hours,” “waiting,” “sitting,” and “lobby” would define a topic which, on inspection, suggests the theme of long wait times. LDA was used to build a topic model using the corpus of review text; afterward, each review was represented as a weighted mixture of the 100 topics generated from the reviews.

### Identifying Differentially Expressed Language Features

Our analysis was aimed at identifying differentially expressed topics in reviews with a 1-star (low) rating versus a 5-star (high) rating considering the bimodal distribution of ratings and based on prior work [11,12]. All statistical analyses were performed in R (version 3.4.1; R Foundation for Statistical Computing). We took a data-driven approach to allow for a more transparent view of the words and phrases that differentiate posts with a high rating (5-star) from those with a low rating (1-star). We isolated the patterns in language topics to obtain correlations in both groups using ordinary least squares (OLS) regression. Treating each review as an observation, OLS regression was performed on standardized LDA derived variables for each review, with the reviews that received 5 stars labeled as 1 and those that received 1 star labeled as 0, and the LDA topic weights of the written review text as the independent variables. Since the variables were standardized, the OLS regression coefficients can be interpreted as Pearson correlations. Topics with a positive coefficient are therefore associated with 5-star reviews, and topics with large negative coefficients are associated with 1-star reviews. We used Bonferroni correction and *P*<.001 for indicating meaningful correlations and the effect size was measured using Pearson *r*. Most highly correlated topics were labeled independently by two coauthors by examining the top 7 terms in each topic. Adjudication of discrepancies occurred via consensus with a third coauthor reviewer.

### Ethical Considerations

The University of Pennsylvania Institutional Review Board deemed the study exempt.

## Results

### Hospital Reviews

We identified 41,095 reviews from 1569 hospitals listed in the American Hospital Association directory with at least one Yelp rating posted from May 2005 to March 2017. Among those hospitals, 919 (59%) had at least one Yelp rating for labor and delivery (median of 9), contributing a total of 6523 labor and delivery reviews about labor and delivery services. The distribution of ratings is shown in Table 2.

### Differentially Expressed Language Features

Themes correlated with favorable ratings included the following: top-notch care (*r*=0.45), expressing gratitude toward staff (*r*=0.41), describing staff as comforting (*r*=0.52), staff having good bedside manner (*r*=0.42), professional and friendly staff (*r*=0.43), the delivery experience (*r*=0.46), modern and clean facilities (*r*=0.44), and hospital food (*r*=0.38; Table 3).

Themes correlated with 1-star labor and delivery reviews included the experience of calling the hospital (*r*=0.33), interactions with reception (*r*=0.31), complaints to management (*r*=0.30), telling others to avoid the hospital (*r*=0.32), a lack of agency among patients (*r*=0.47), and issues with discharging from the hospital (*r*=0.32; Table 4).

**Table 3:** Yelp differential language analysis topics associated with positive (5-star) labor and delivery reviews.

Yelp domain (determined by Yelp) and Yelp topic (topic terms)		Correlation, Pearson *r*	Example quote
**Positive experience**			
	Top-notch care (excellent, received, care, top, notch, wonderful, attentive, amazing)	0.448	Thank you to the ENTIRE [hospital name] Pediatric unit. They have taken EXCELLENT care of our baby. Your attention and dedication was top notch. We greatly appreciate it....
**Compassionate caring staff**			
	Grateful (amazing, team, wonderful, grateful, life, love, god, team, saved, bless, remember)	0.405	We are very blessed to have such an amazing team of nurses. The support and caring was priceless. We most likely will have baby 2 in here. If you looking for a happy medium between home-birth or hospital-birth, this place is the answer.
	Comforting (made, comfortable, feel, helpful, questions, friendly, caring, pleasant, attentive)	0.521	Both of my daughters gave birth at [the hospital] and raved about the care they got. The nursing staff is compassionate and very skilled!
	Bedside manner (great, bedside, dr, manner, awesome, kind, fantastic, sweet, tech, compassionate)	0.416	Delivered my first here. Clean, friendly, knowledgeable staff. Very attentive in Labor and Delivery and until we went home. Made the week long stay as comfortable as possible. Nurses had really good bedside manner. I would recommend this hospital to others.
	Professional/friendly staff (friendly, staff, professional, helpful, efficient, service, recommend, highly, courteous)	0.426	Excellent labor/delivery and postpartum experience. Every nurse we encountered was kind and caring. Highly recommended.
**Clinical service**			
	Delivery experience (delivery, labor, baby, amazing, birth, wonderful, maternity, nicu^a^, ward, helpful)	0.462	Maternity ward was awesome when I delivered my baby in October everyone was exceptional.
**Facilities and amenities**			
	Modern and clean (facility, rooms, clean, friendly, helpful, nice, super, equipment, modern, beautiful)	0.444	This is hospital is one of the cleanest around! The maternity staff is excellent and so are the facilities!
	Hospital food (food, pretty, order, stay, cafeteria, menu, private, dinner, free)	0.380	I delivered my daughter here and absolutely love everything about this hospital from the warm staff, comfortable rooms and amazing kitchen menu for patient meals.

^a^NICU: neonatal intensive care unit.

**Table 4:** Yelp differential language analysis topics associated with negative (1-star) labor and delivery reviews.

Yelp domain (determined by Yelp) and Yelp topic (topic terms)		Correlation, Pearson *r*	Example quote
**Negative interactions**			
	Lack of agency (told, asked, didn’t, questions, wasn’t, couldn’t, rude, talk, telling, upset)	0.465	This review is for the prenatal clinic. I called this morning because I had been advised to do so by my Dr. The lady who answered the phone was extremely rude and unprofessional. Her exact words “ and your calling us for????” With the rudest tone I have ever heard from a healthcare professional. I was beyond shocked and will never go to their clinic.
	Being discharged (information, discharge, medications, papers, husband, refused, stated, physician, attending, signed)	0.321	Beware!!! Don't entrust this incompetent facility with your life!! Intake form - misspelling of name and incorrect recording of birth date so records could not be found until 4phone calls later…
**Communication with hospital**			
	Calls (phone, call, number, person, message, answer, hold, office, transferred)	0.326	Horrible customer service. Called the operator to inquire about setting up a prenatal appointment to find an OBGYN and she told me to google them to find one I like! Ha what a joke.
	Reception (desk, front, asked, check, walked, minutes, arrived, paperwork)	0.305	Maternity receptionist is still rude as hell. Wife sent over by office for emergency monitoring.... She told us to wait while she finished putting stickers on a folder.
	Complaints to management (complaint, manager, response, letter, lack, contact, advocate, concerns, case, report)	0.296	I had a baby at [hospital name] in 2008. Horrible, scary, TRAUMATIC experience. Incompetence, unprofessionalism, and bad medicine…In spite of numerous conversations with various individuals at the hospital, and a lengthy grievance filed against the hospital with my insurance, no one at the hospital ever said “I'm sorry.”
**Wait times**			
	Long wait (hours, waited, hour, finally, worst, sitting, triage, ridiculous)	0.409	Anyone giving this place 5 stars works here. Longest wait ever over 2 hours with a ectopic pregnancy. This place is horrible
**Pain meds**			
	Medication (pain, meds, gave, prescription, morphine, prescribed, migraine, severe)	0.325	If you want to be constantly asked if you’re a drug addict while you cry in pain this is your place. Even at 43 under a Sutter West doctors care, had a baby there 3 months earlier have 9 years of [hospital name] medical records, and that still didn't help the staff treat not me like an addict until my x-Rays came back then it's like I'm so sorry here's some pills have a nice day call your doctor. :(
**Would not recommend**			
	Unprofessional staff (worst, rude, terrible, awful, avoid, incompetent, horrible, place, unprofessional)	0.315	This Hospital is one of the worst hospitals in the [county name] county. Their staff is very rude. They almost killed my mother during child birth due to uneducated staff and negligence. DO NOT GO HERE! Not safe.
**Specialized medical care**			
	Tests (test, results, sample, urine, lab, ordered, UTI^a^, negative)	0.342	I came in for my 8week old baby he had a temp. Of 101F I came in at 11pm and now it's 4:15 am we are still waiting on some results I know it does not take that long for something to come back about my baby knowing the baby haves a high temp.
	IV^b^ (arm, hand, needle, IV, put, started, painful, stop, screaming, catheter, veins)	0.339	Never ever would I bring my baby to this hospital walked in my room and found a needle on the floor
	Kidney stones (kidney, pain, abdominal, severe, stomach, stone, meds, excruciating)	0.315	I went in for serious stomach pains while I was pregnant, the doctor said I had gall stones that needed to be removed via surgery, scheduled surgery for after my baby was born just to find out later that there was no gall stones, never did find out what sent me to the emergency room in debilitating pain while I was pregnant!

^a^UTI: urinary tract infection.

^b^IV: intravenous.

## Discussion

### Principal Findings

This study found identifiable themes associated with high and low ratings, offering insights into what patients seeking labor and delivery services care about most. Online reviews about hospitals include comments about the experience of labor and delivery care. Although online reviews are not validated and may attract or amplify the most negative comments [13,16], they reflect raw reports from patients unconstrained by pre-established topics.

Positive reviews on the labor and delivery experience overwhelmingly cited compassionate and attentive hospital staff. Nurses were frequently cited as the most important component of the experience. For 5-star reviewers who criticized their experience in any way, caring and helpful nurses and staff almost always made up for the negative aspects of their stay. In addition, 5-star reviews in our study largely referenced positive feelings about hospital staff and the importance of hospital amenities (often citing spa showers, advanced technology, and appealing decor). Prior work reported that, in the patient-provider relationship in an obstetrics and gynecology setting, patients reported greater satisfaction with their health care experience when they had a positive relationship with their care team, which parallels our finding that patients are more satisfied when providers are caring and attentive [17]. Compassion of staff is not a topic measured in HCAHPS surveys. Additionally, HCAHPS and Press Ganey do not include free-text questions; rather, questions are multiple choice.

Negative reviews of labor and delivery included topics typically inverse to the topics discussed in positive reviews. Raters cited negative interactions and lack of communication with hospital staff, long wait times, and low-quality obstetrics care in 1-star labor and delivery reviews. In a prior review of patient satisfaction in obstetrics care, researchers interviewed patients and compiled a total of 51 items related to patient satisfaction [18]. The list included multiple characteristics related to provider communication style, including compassion/sensitivity, communication, accessibility, support, and positive affirmation of birthing process. Access to and communication with hospital staff contribute to a more positive patient experience in the context of labor and delivery care. Understanding the common themes of positive and negative experiences may help clinical and operational staff create initiatives and protocols that lead to better patient encounters.

### Limitations

This study has several limitations. The American Hospital Association data set represents broader obstetrical programs (eg, the Hospital of the University of Pennsylvania) but may miss subsidiary programs (eg, Penn Ob/Gyn & Midwifery Care). The bimodal distribution of reviews may amplify the voices of those with strongly positive and strongly negative experiences, muting the more nuanced and mixed experiences. Clinical terms and procedures may be talked about in slang and ways that are harder to identify using automated techniques. However, using machine learning techniques allows for the analysis of hundreds of thousands of reviews as opposed to what is possible with human coders. In addition, Yelp reviews are not validated and may vary in quality and quantity. To counter this, we eliminated reviews “not recommended” by Yelp (a measure indicating a review is likely to be fake). “Not recommended” reviews are determined automatically by Yelp’s proprietary algorithm that considers a number of factors to try and remove fake reviews (eg, one person posting many reviews from the same computer). In the future, including other online review platforms may provide richer insights. The practical application of this data is largely valuable as a supplemental insight into the patient’s psychological experience of their labor and delivery care. Understanding the themes that correlate to high and low reviews may provide a place to start when developing standardized surveys for measuring care.

### Conclusions

Transparency of hospital performance data is vital to enhancing patient trust and improving health care delivery. Online rating websites may help foster trust and goodwill between hospitals and their consumers, allow consumers to make more informed decisions, and encourage quality improvements [16,19]. Increasing the validity and scientific rigor of these narrative feedback platforms may increase the value of these patient narratives for further improving obstetrics care in the United States [20,21].

## Abbreviations

HCAHPS: Hospital Consumer Assessment of Healthcare Providers and Systems
LDA: latent Dirichlet allocation
OLS: ordinary least squares

## References

[1] Zusman EE (2012). HCAHPS replaces Press Ganey survey as quality measure for patient hospital experience. Neurosurgery.

[2] Ranard BL, Werner RM, Antanavicius T, Schwartz HA, Smith RJ, Meisel ZF, Asch DA, Ungar LH, Merchant RM (2016). Yelp Reviews Of Hospital Care Can Supplement And Inform Traditional Surveys Of The Patient Experience Of Care. Health Aff (Millwood).

[3] Jordan H, White A, Joseph C Costs and benefits of HCAHPS: final report. Abt Assoc Inc.

[4] (2015). The HCAHPS survey - frequently asked questions.

[5] O'Malley AJ, Zaslavsky A, Hays R, Hepner KA, Keller S, Cleary PD (2005). Exploratory factor analyses of the CAHPS Hospital Pilot Survey responses across and within medical, surgical, and obstetric services. Health Serv Res.

[6] Bardach NS, Asteria-Peñaloza R, Boscardin WJ, Dudley RA (2013). The relationship between commercial website ratings and traditional hospital performance measures in the USA. BMJ Qual Saf.

[7] Emmert M, Meszmer N, Sander U (2016). Do Health Care Providers Use Online Patient Ratings to Improve the Quality of Care? Results From an Online-Based Cross-Sectional Study. J Med Internet Res.

[8] Bardach NS, Lyndon A, Asteria-Peñaloza R, Goldman LE, Lin GA, Dudley RA (2016). From the closest observers of patient care: a thematic analysis of online narrative reviews of hospitals. BMJ Qual Saf.

[9] Kilaru AS, Meisel ZF, Paciotti B, Ha YP, Smith RJ, Ranard BL, Merchant RM (2016). What do patients say about emergency departments in online reviews? A qualitative study. BMJ Qual Saf.

[10] Woods KD (2015). Search Your Heart: What Press Ganey Can Never Measure. Obstet Gynecol.

[11] Agarwal AK, Pelullo AP, Merchant RM (2019). "Told": the Word Most Correlated to Negative Online Hospital Reviews. J Gen Intern Med.

[12] Agarwal AK, Mahoney K, Lanza AL, Klinger EV, Asch DA, Fausti N, Tufts C, Ungar L, Merchant RM (2019). Online Ratings of the Patient Experience: Emergency Departments Versus Urgent Care Centers. Ann Emerg Med.

[13] Merchant RM, Volpp KG, Asch DA (2016). Learning by Listening-Improving Health Care in the Era of Yelp. JAMA.

[14] McCallum AK Mallet: A machine learning for language toolkit. MALLET.

[15] Blei D, Ng A, Jordan M (2003). Latent Dirichlet Allocation. J Mach Learn Res.

[16] Lee V (2017). Transparency and Trust — Online Patient Reviews of Physicians. N Engl J Med.

[17] Di Blasi Z, Harkness E, Ernst E, Georgiou A, Kleijnen J (2001). Influence of context effects on health outcomes: a systematic review. Lancet.

[18] Howell EA, Concato J (2004). Obstetric patient satisfaction: asking patients what they like. Am J Obstet Gynecol.

[19] Lagu T, Metayer K, Moran M, Ortiz L, Priya A, Goff SL, Lindenauer PK (2017). Website Characteristics and Physician Reviews on Commercial Physician-Rating Websites. JAMA.

[20] Cognetta-Rieke C, Guney S (2014). Analytical Insights from Patient Narratives: The Next Step for Better Patient Experience. J Patient Exp.

[21] Schlesinger M, Grob R, Shaller D, Martino SC, Parker AM, Finucane ML, Cerully JL, Rybowski L (2015). Taking Patients' Narratives about Clinicians from Anecdote to Science. N Engl J Med.

